# Hyperbilirubinemia Maintained by Chronic Supplementation of Unconjugated Bilirubin Improves the Clinical Course of Experimental Autoimmune Arthritis

**DOI:** 10.3390/ijms22168662

**Published:** 2021-08-12

**Authors:** Tomas Sykora, Pavel Babal, Kristina Mikus-Kuracinova, Frantisek Drafi, Silvester Ponist, Monika Dvorakova, Pavol Janega, Katarina Bauerova

**Affiliations:** 1 Department of Pathology, Faculty of Medicine, Comenius University in Bratislava, Spitalska 24, 81372 Bratislava, Slovakia; pavel.babal@fmed.uniba.sk (P.B.); kristina.kuracinova@fmed.uniba.sk (K.M.-K.); pavol.janega@fmed.uniba.sk (P.J.); 2 Centre of Experimental Medicine, Institute of Experimental Pharmacology and Toxicology, Slovak Academy of Sciences, Dúbravská Cesta 9, 84104 Bratislava, Slovakia; frantisek.drafi@savba.sk (F.D.); exfasipo@savba.sk (S.P.); exfakbau@savba.sk (K.B.); 3 Department of Medical Chemistry, Biochemistry and Clinical Biochemistry, Faculty of Medicine, Comenius University in Bratislava, Spitalska 24, 81372 Bratislava, Slovakia; monika.dvorakova@fmed.uniba.sk

**Keywords:** adjuvant-induced arthritis, bilirubin, immunomodulation, inflammation, white blood cells

## Abstract

Rheumatoid arthritis (RA) is a chronic multisystem disease, therapy of which remains a challenge for basic research. The present work examined the effect of unconjugated bilirubin (UCB) administration in adjuvant-induced arthritis (AIA)—an experimental model, in which oxidative stress (OS), inflammation and inadequate immune response are often similar to RA. Male Lewis rats were randomized into groups: CO—control, AIA—untreated adjuvant-induced arthritis, AIA-BIL—adjuvant-induced arthritis administrated UCB, CO-BIL—control with administrated UCB. UCB was administered *intraperitoneally* 200 mg/kg of body weight daily from 14th day of the experiment, when clinical signs of the disease are fully manifested, to 28th day, the end of the experiment. AIA was induced by a single intradermal immunization at the base of the tail with suspension of *Mycobacterium butyricum* in incomplete Freund’s adjuvant. Clinical, hematologic, biochemical and histologic examinations were performed. UCB administration to animals with AIA lead to a significant decrease in hind paws volume, plasma levels of C-reactive protein (CRP) and ceruloplasmin, drop of leukocytes, lymphocytes, erythrocytes, hemoglobin and an increase in platelet count. UCB administration caused significantly lowered oxidative damage to DNA in arthritic animals, whereas in healthy controls it induced considerable oxidative damage to DNA. UCB administration also induced atrophy of the spleen and thymus in AIA and CO animals comparing to untreated animals. Histological signs of joint damage assessed by neutrophils infiltration and deposition of fibrin were significantly reduced by UCB administration. The effects of exogenously administered UCB to the animals with adjuvant-induced arthritis might be identified as therapeutic, in contrast to the effects of UCB administration in healthy animals rather classified as toxic.

## 1. Introduction

Rheumatoid arthritis (RA) is a complex inflammatory disease of joints, which typically affect small joints of the hand and feet. If left untreated, it leads to a progressive joint cartilage destruction and disability. Moreover, this disease can damage a wide variety of body systems, including the skin, eyes, lungs, heart and blood vessels. The prevalence of RA in population is around 1% [1]. RA is predominantly classified on the basis of the clinical phenotype, autoantibody production, influenced by both genetic and environmental factors [2,3]. Joint synovium is normally a structure with low cellularity and a delicate intimal lining. Activation of signal pathways of cytokines and other inflammation markers lead to infiltration of the synovium by CD4^+^ T-cells, B-cells, macrophages and neutrophils [4,5]. Increased numbers of macrophage-like and fibroblast-like synoviocytes result in synovial hyperplasia. Locally released degradation enzymes, including metalloproteinases, serine proteases and aggrecanases, digest the extracellular matrix and destroy the articular structures. T-cell activation is part of the process but the reason of systemic loss of tolerance turning to a localized onset of inflammation in the joint is still unclear [6].

According to Smolen et al. (2014) the treatment for RA can be classified into biological original and biosimilar disease-modifying anti-rheumatic drugs (boDMARDs and bsDMARDs, respectively) and the former non-biologic DMARDs into conventional synthetic and targeted synthetic DMARDs (csDMARDs and tsDMARDs, respectively) [7]. Methotrexate (MTX) belongs to the csDMARDs and is widely used as the first-line therapy in RA [8,9]. Recent advances in RA treatment include the application of approved novel agents inhibiting IL-6, IL-17 (boDMARDs) and Janus kinase (tsDMARDs) as well as biosimilars (bsDMARDs) [6,10,11]. Although there is a number of RA treatment alternatives, many of the approved agents are not available for most of the patient and side effects as well as treatment resistance are limiting the best treatment option. Despite of advances in drug development for RA, a group of patients may benefit of alternative treatments such as antioxidant intake in form of fresh fruits/vegetables as well as food supplements [12].

Nearly a century ago, jaundiced patients were observed to have surprising and spontaneous remissions from incurable immunologic diseases including rheumatoid arthritis, allergy, and asthma. The mystery of why this phenomenon occurred remains unresolved to this day [13]. Bilirubin is a secondary breakdown product of normal heme catabolism that is excreted in bile and urine, and elevated levels may indicate certain diseases [14]. For decades was bilirubin considered as a metabolism side product with no specific purpose but recent data indicate that bilirubin exhibits potent antioxidant properties with positive clinical consequence and relatively low toxicity [15,16,17]. Most recently, the molecule has been found to possess immunomodulatory properties that rival its redox capacity, possibly explaining its ability to suppress inflammation [13]. Higher serum levels of bilirubin are linked with an absence of inflammatory diseases such as RA [16,18]. Gilbert’s syndrome may be defined as harmless unconjugated hyperbilirubinemia due to a decreased conjugation enzymatic capacity in liver [19]. It is known that this trait in people may reduce health risks in cardiovascular diseases [15,20,21].

Unconjugated bilirubin (UCB) investigation on the adjuvant-induced arthritis (AIA) model has not been performed yet. In the presented experimental study we evaluate the tissue protective and immunomodulatory effect of UCB on the course of AIA where oxidative stress, inflammation and inadequate immune response lead to development of significant pathology [22]. In our pre-clinical research, there was an interest to find out which of the markers are modified in AIA conditions and to compare this with treated healthy controls. We have described the anti-inflammatory properties of UCB for the first time, UCB has furthermore exhibited its properties on many other markers on the AIA model. Further research of UCB may introduce interest for development of its new pharmaceutical forms to improve therapeutic outcome of RA treatment.

## 2. Results

### 2.1. Bilirubin Levels

Serum levels of total and conjugated bilirubin were measured on 28th day of the experiment. Values of total bilirubin in control group (CO) and AIA, 0.20 ± 0.07 and 0.29 ± 0.11 µM/L, are naturally very low in Lewis rats and conjugated bilirubin in these two groups was undetectable [23]. UCB administration increased its levels approximately hundred times. In adjuvant-induced arthritis group with UCB administration (AIA-BIL) was the average level of conjugated bilirubin 4.95 ± 0.39 µM/L and of total bilirubin 28.67 ± 2.23 µM/L. In control group with UCB administration (CO-BIL) was the average level of conjugated bilirubin 5.93 ± 1.35 µM/L and of total bilirubin 29.73 ± 1.82 µM/L.

### 2.2. Changes in Hind Paw Volume and Body Weight

**Figure 1:**
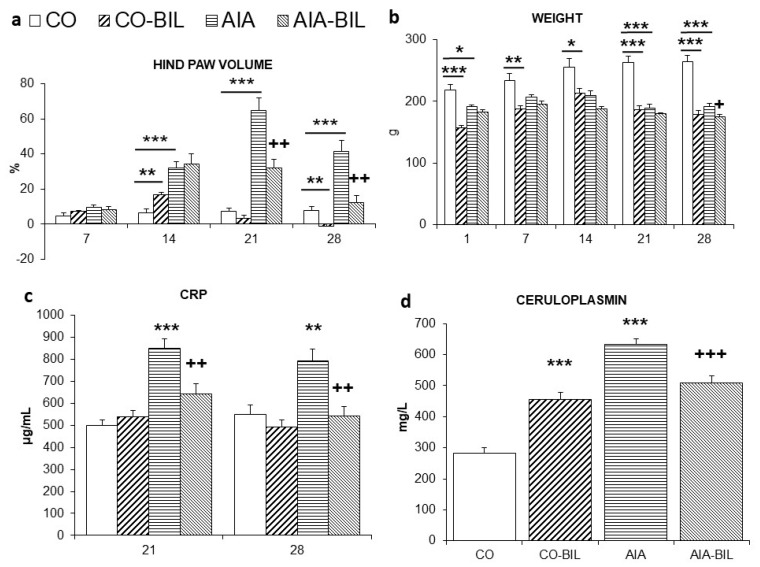
(**a**). Time profile of hind paw volume changes during the course of the experiment. (**b**). Body weight changes of the animals during the experiment. (**c**). CRP levels change on 21st and 28th day and (**d**). Ceruloplasmin levels at the end of the experiment. CO—control group, CO-BIL—control group administered with UCB 200 mg/kg of body weight daily i.p. from day 14, AIA—group with adjuvant induced arthritis, AIA-BIL—group with adjuvant induced arthritis administered with UCB 200 mg/kg of body weight daily i.p. from day 14. Results are expressed as mean ± SEM, *n* = 7–8. Significant difference between groups CO and CO-BIL, CO and AIA: *** *p* < 0.001 vs. CO, ** *p* < 0.01 vs. CO, * *p* < 0.05 vs. CO. Significant difference between groups AIA and AIA-BIL: ^+++^ *p* < 0.001 vs. AIA, ^++^ *p* < 0.01 vs. AIA, ^+^ *p* < 0.05 vs. AIA.

Swelling of the hind paws was a marker of clinical progression [24]. Induction of AIA resulted in significant swelling of the hind paw joints evaluated as the change in hind paw volume (HPV, *p* < 0.001 vs. CO on day 14, 21 and 28). In the AIA-BIL group, UCB administration lead to a decrease of HPV compared to untreated AIA group (*p* < 0.01 vs. AIA on day 21 and 28; Figure 1a), which improved mobility of the animals. In CO-BIL was HPV on day 28 significantly decreased comparing to healthy CO animals (*p* < 0.01 vs. CO on day 28; Figure 1a). There was observed a general body weight loss of the animals with AIA and in both groups of animals administered UCB since day 14 towards the end of the experiment (for CO-BIL *p* < 0.001 vs. CO on day 21 and 28, for AIA-BIL *p* < 0.05 vs. AIA on day 28; Figure 1b).

### 2.3. Changes in Blood Screen

**Figure 2:**
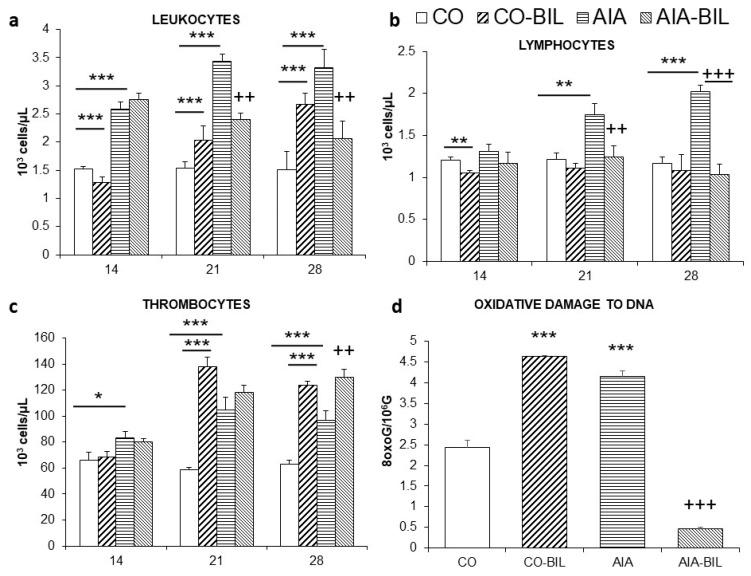
Time profile of changes in (**a**). leukocytes, (**b**). lymphocytes and (**c**). thrombocytes (**d**). Oxidative damage to DNA evaluated on day 28 of the experiment CO—control group, CO-BIL—control group administered with UCB 200 mg/kg of body weight daily i.p. from day 14, AIA—group with adjuvant induced arthritis, AIA-BIL—group with adjuvant induced arthritis administered with UCB 200 mg/kg of body weight daily i.p. from day 14. Results are expressed as mean ± SEM, n = 7–8. Significant difference between groups CO and CO-BIL, CO and AIA: *** *p* < 0.001 vs. CO, ** *p* < 0.01 vs. CO, * *p* < 0.05 vs. CO. Significant difference between groups AIA and AIA-BIL: ^+++^ *p* < 0.001 vs. AIA, ^++^ *p* < 0.01 vs. AIA.

Induction of AIA lead in AIA animals to an increase in leukocyte, lymphocyte and platelet counts in the peripheral blood (*p* < 0.001 vs. CO on day 14, 21 and 28; Figure 2a–c respectively). UCB administration caused a significant decrease of leukocytes (*p* < 0.01) and lymphocytes (*p* < 0.01) that continued towards the end of the experiment (Figure 2a,b). UCB administration produced significant changes in the number of red blood cells, in levels of hemoglobin and in the mean corpuscular volume (MCV) in AIA animals. In control animals administration of UCB caused significant decrease of hemoglobin level and significant decrease in MCV, thus the size of erythrocytes was smaller (Table 1). On the contrary, as demonstrated in both AIA-BIL and CO-BIL groups, UCB administration caused significant elevation of thrombocytes when compared to AIA and CO, respectively (Figure 2c).

### 2.4. Markers of Inflammation and Organ Weight

Administration of UCB caused in AIA-BIL significant decrease of CRP (*p* < 0.01 vs. AIA on day 21 and 28; Figure 1c) and ceruloplasmin (*p* < 0.001 vs. AIA on day 28; Figure 1d) in AIA-BIL compared to AIA, which corresponded with the clinical course of the disease in the experimental animals.

**Figure 3:**
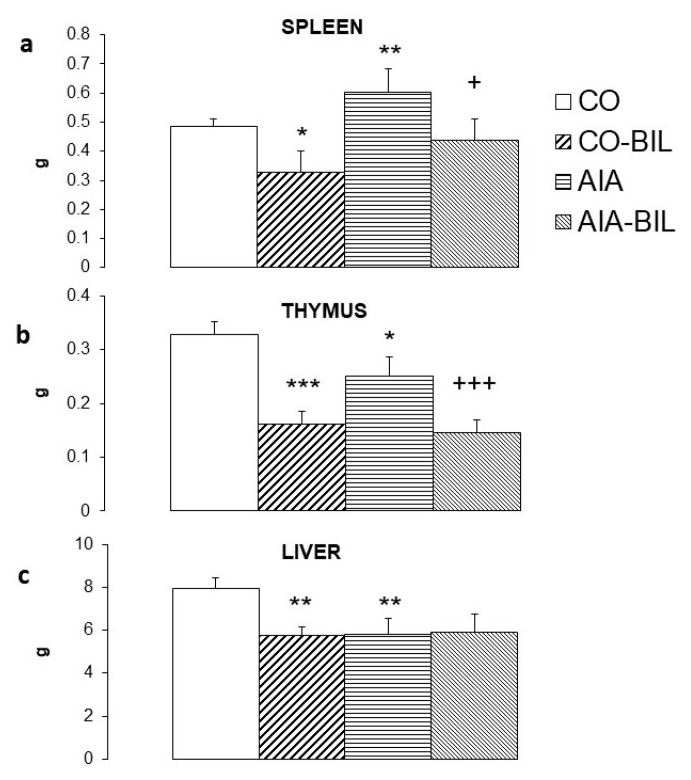
Weight of selected organs at the end of the experiment; (**a**). spleen, (**b**). thymus, (**c**). liver. CO—control group, CO-BIL—control group administered with UCB 200 mg/kg of body weight daily i.p. from day 14, AIA—group with adjuvant induced arthritis, AIA-BIL—group with adjuvant induced arthritis administered with UCB 200 mg/kg of body weight daily i.p. from day 14. Results are expressed as mean ± SEM, n = 7–8. Significant difference between groups CO and CO-BIL, CO and AIA: *** *p* < 0.001 vs. CO, ** *p* < 0.01 vs. CO, * *p* < 0.05 vs. CO. Significant difference between groups AIA and AIA-BIL: ^+++^ *p* < 0.001 vs. AIA, ^+^ *p* < 0.05 vs. AIA.

At the end of the experiment, liver, thymus and spleen were collected and weighted. Animals with UCB administration had significantly smaller spleen (CO-BIL *p* < 0.05 vs. CO, AIA-BIL *p* < 0.05 vs. AIA; Figure 3a) and thymus (CO-BIL *p* < 0.001 vs. CO; AIA-BIL *p* < 0.001 vs. AIA; Figure 1b), liver was significantly smaller in CO-BIL comparing to CO (*p* < 0.01; Figure 1b), but about the same weight in AIA and AIA-BIL (Figure 3).

### 2.5. Oxidative Damage to DNA and Histological Examination

Oxidative damage to DNA evaluated at the end of the experiment was significantly increased in the AIA and dramatically dropped by bilirubin in the AIA-BIL group (*p* < 0.001; Figure 2d). UCB administration in CO-BIL resulted in a significantly higher oxidative DNA damage comparing to CO group (*p* < 0.001; Figure 2d).

**Figure 4:**
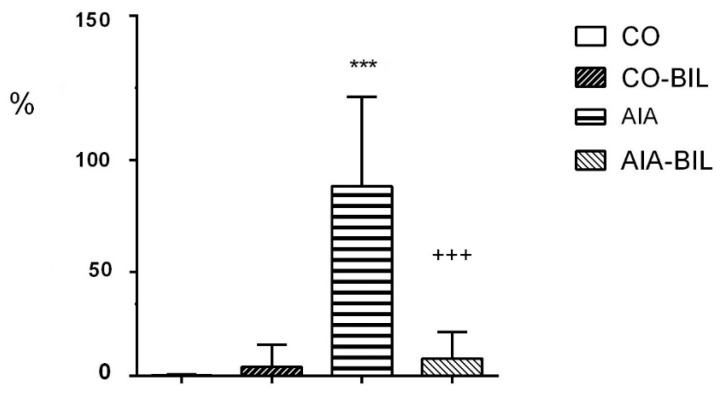
Infiltration of joints by granulocytes. CO—control group, CO-BIL—control group administered with UCB 200 mg/kg of body weight daily i.p. from day 14, AIA—group with adjuvant induced arthritis, AIA-BIL—group with adjuvant induced arthritis administered with UCB 200 mg/kg of body weight daily i.p. from day 14. Results are expressed as mean ± SEM, n = 7–8. Significant difference between groups CO and CO-BIL, CO and AIA: *** *p* < 0.001 vs. CO. Significant difference between groups AIA and AIA-BIL: ^+++^ *p* < 0.001 vs. AIA.

**Figure 5:**
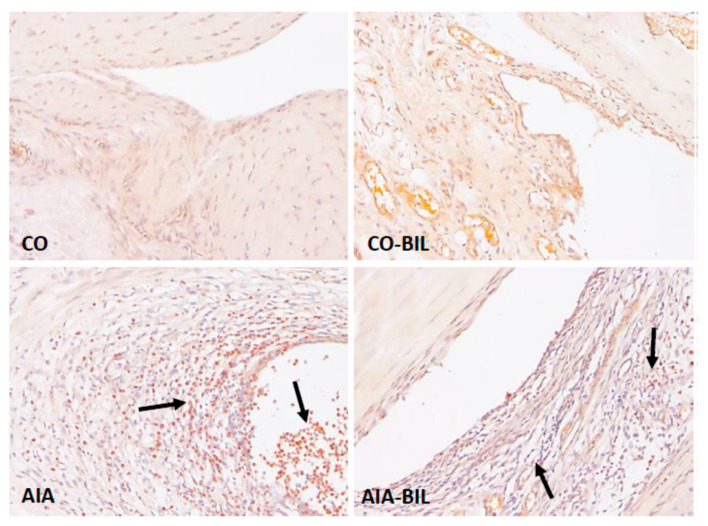
Infiltration of joints by granulocytes detected with naphtol-AS-D-chloroacetate esterase (CHAE) activity. Dense granulocytic infiltrate (arrow) in the inflamed joints (AIA) was reduced by UCB treatment (AIA-BIL). CO—control group, CO-BIL—control group administered with UCB 200 mg/kg of body weight daily i.p. from day 14, AIA—group with adjuvant induced arthritis, AIA-BIL—group with adjuvant induced arthritis administered with UCB 200 mg/kg of body weight daily i.p. from day 14. CHAE, 100×.

**Figure 6:**
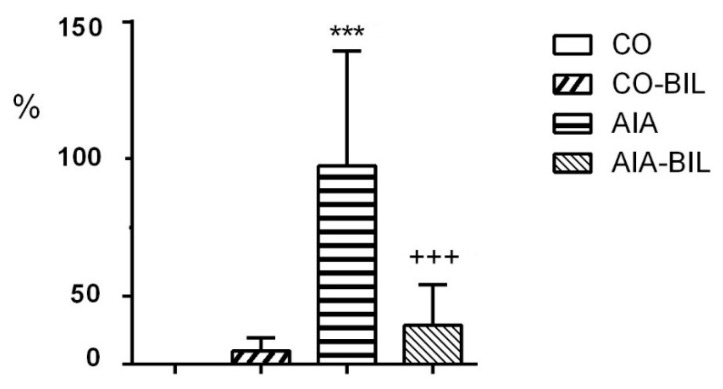
Fibrin exudation into joints. CO—control group, CO-BIL—control group administered with UCB 200 mg/kg of body weight daily i.p. from day 14, AIA—group with adjuvant induced arthritis, AIA-BIL—group with adjuvant induced arthritis administered with UCB 200 mg/kg of body weight daily i.p. from day 14. Results are expressed as mean ± SEM, n = 7–8. Significant difference between groups CO and CO-BIL, CO and AIA: *** *p* < 0.001 vs. CO. Significant difference between groups AIA and AIA-BIL: ^+++^ *p* < 0.001 vs. AIA.

**Figure 7:**
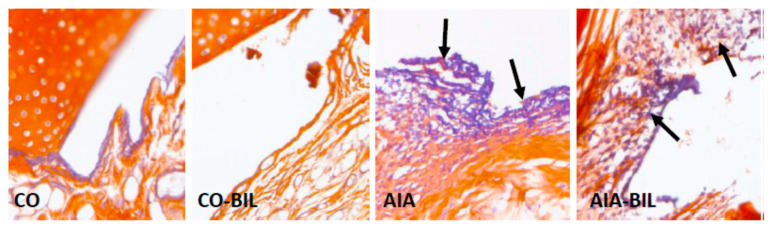
Detection of fibrin in joints with Mallory’s phosphotungstic acid hematoxylin (PTAH) staining. Massive exudation of fibrin (arrow pointing at blue color) in the inflamed joints (AIA) was reduced by UCB treatment (AIA-BIL). CO—control group, CO-BIL—control group administered with UCB 200 mg/kg of body weight daily i.p. from day 14, AIA—group with adjuvant induced arthritis, AIA-BIL—group with adjuvant induced arthritis administered with UCB 200 mg/kg of body weight daily i.p. from day 14. PTAH, 200×.

Bilirubin treatment dramatically reduced the inflammatory cell infiltrate in AIA-BIL (*p* < 0.001 vs. AIA; Figure 4). Histological evaluation of the hind paw knee joints in animals with arthritis showed enlargement of the articular cavity and with leukocyte accumulation, interstitial edema and infiltration of the soft tissues by leukocytes. Joint inflammation was documented by dense infiltration of the periarticular tissues by neutrophilic granulocytes and their presence in the articular cavity, as demonstrated with chloroacetylesterase (CHAE) activity (Figure 5). The fibrin exudation was significantly reduced in the AIA-BIL group (*p* < 0.001 vs. AIA; Figure 6). Fibrin exudation detected by phosphotungstic acid hematoxylin (PTAH) staining, as the sign of an active inflammatory process, was in high amounts present in the joints in all AIA samples (Figure 7).

## 3. Discussion

The aim of the study was to observe changes in the course of an experimentally-induced arthritis disease in the settings of unconjugated bilirubin administration. The model of AIA was chosen as a disease similar to human RA that allows to monitor progression of the disease and effects of the experimental treatment [25]. The murine models have many limitations in regard to human RA, such as the genetically-based onset of RA and the agent of disease model induction is known. However, our research team is focusing on the symptoms mitigation as well as other markers of inflammation that might be relevant for further RA pharmacotherapy, but strictly on the preclinical research level. Bilirubin presence in plasma is natural in humans, but in rats its levels are naturally low, almost undetectable in the physiological state [23], thus, the model allows to study the specific effect of UCB administration. UCB’s impact on function of the immune system was described in several different experimental settings [23,26]. To our knowledge, this is the first study that documents complex changes of blood elements variables in the time course during high dose of UCB administration for the period of 14 days (Figure 2a–c, Table 1).

Adjuvant induced arthritis brought about inflammatory changes in the hind paw knee joints clinically manifested by swelling, as previously demonstrated [25]. Inflammation in hind paw joints is caused by granulocyte infiltration leading to fibrin depositions as seen in our experiments (Figure 4, Figure 5, Figure 6 and Figure 7) [27,28]. Decrease of the hind paws volume, the reduction of leukocytes, granulocyte infiltration and fibrin deposition in the joints histologic evaluation proved significant effects of UCB administration on the course of the disease (Figure 1a, Figure 2a, Figure 4, Figure 5, Figure 6 and Figure 7). Therapeutic use of UCB has not been used before in an experimental model of RA. Bonneli (2012) used UCB precursor—biliverdin as a heme oxygenase-1 end product, which successfully improved the course of murine collagen induced arthritis (CIA). Histological examination of affected joints in mice after 60 days long treatment showed lowered inflammation and bone destruction in the CIA mouse model [29]. Epidemiological study National Health and Nutrition Examination Survey by Fischman (2010) concluded that higher total bilirubin levels were linked to a reduced risk of RA and as a protective factor in humans [16]. These data, supported also by the results of our experimental findings, indicate the protective role of bilirubin administration, and also its pathological consequences.

The plausible anti-inflammatory effects of unconjugated bilirubin in our experiment were supported also by decreased levels of inflammation proteins such as CRP and ceruloplasmin (Figure 1c,d). The relationship between bilirubin and CRP and suppressing effect of bilirubin on CRP levels have already been observed in humans [30]. It has also been documented as in vitro experiment by Khan and Poduval (2011) [26], and as in vivo experiment in a mouse model of endotoxemia [31], but correlation between hyperbilirubinemia and low levels of ceruloplasmin was only part of the clinical assessment in full-term newborn infants [32].

Immunomodulative properties of UCB were part of various animal studies [13]. In general, administration of UCB showed major impact on the immune system. Experiments performed in vitro documented toxic effect of 25 µM/L concentration of UCB on unfractionated splenocytes and splenic T cells, B cells, macrophages and LPS-stimulated CD19^+^ B cells [26]. In vivo administration of UCB to healthy animals induced atrophy of the spleen, depletion of bone marrow cells, peripheral leukopenia and decreased lymphocyte count [26]. High levels of UCB improved the outcome of experimental autoimmune encephalomyelitis. The immunomodulatory effect of UCB was attributed to possible induction of apoptosis in reactive T cells [23]. The above-mentioned findings resulted from different study designs. In our experimental setting, the spleen, the thymus and the liver were assessed by means of the change of their weight. Except for the liver, we observed atrophy of the thymus and the spleen in the AIA group caused by UCB administration (Figure 3). In addition to the evaluation of the impact of UCB on the arthritis clinical presentation changes, our data also recorded changes in the blood screen in the period of 14 days. In the experiment we showed that increased levels of UCB caused a universal decrease in the number of lymphocytes, atrophy of the spleen, thymus and the liver in both, the AIA and the CO animals (Figure 2b and Figure 3). All these findings point at the immunomodulatory effect of high levels of UCB and with positive effect on the course of AIA.

UCB administration to AIA-BIL group deepened atrophy of the thymus in comparison with untreated AIA group (Figure 3b). Similar observation of the thymic regression as a response to the outflow of mature T lymphocytes in the context of inflammatory cells redistribution in the AIA experimental model has already been reported [25]. Analyzing the effect of UCB on the spleen, UCB administration to AIA-BIL restore the hypertrophy to the size of untreated CO group (Figure 3a). Due to high leukocytosis and the ongoing inflammation in the AIA animals, hypertrophy of the spleen may be the result. Administration of UCB in the AIA group lead to a greater thymus atrophy and a significant decrease of weight of the spleen. These results show similar course of the atrophy in the thymus and the spleen in the CO-BIL group induced by administration of UCB, which confirms the findings previously reported by others [26].

General impact of UCB on hematopoiesis is demonstrated by changes of platelets and erythrocytes parameters (Figure 2c, Table 1). The lifespan of erythrocytes in rats is approximately 60 days [33], which might explain the delayed decrease compared to the rapid changes in leukocytes. The drop of hemoglobin caused by hyperbilirubinemia has already been described by others [26]. Brito (2006), according to the observation of hyperbilirubinemic and normobilirubinemic neonates, presumed toxicity of UCB to erythrocyte’s membrane [34]. In contrast to this assumption, McDonagh (2007) claimed that Gunn rats and patients with Crigler-Najjar syndrome have persistent unconjugated hyperbilirubinemia and do not suffer from extensive hemolysis [35]. Of particular interest in our experiment is the observed rise in thrombocytes after UCB administration, both in healthy and the AIA animals (Figure 2c). This is a unique observation that has not been described before. The underlying mechanism for rising numbers of platelets might be linked with the atrophy of the spleen resulting in decreased platelet removal from circulation and older platelets being allowed to circulate for a longer period of time. These suggestions will require to be confirmed by further research.

Bilirubin is known to possess powerful antioxidant properties [36,37]. Oxidative stress is an important factor of inflammation and plays an important role in the pathogenesis of autoimmune diseases such as RA and its experimental model—adjuvant induced arthritis [38]. This hypothesis was confirmed also by our results, where administration of UCB significantly decreased oxidative DNA damage in sick animals (Figure 2d). On the other hand, the results in the control group were remarkably completely the opposite. Administration of UCB caused in healthy animals a massive oxidative damage to DNA (Figure 2d). Similar results are obtained from experiments on Gunn rats that suffer naturally from unconjugated hyperbilirubinemia. The DNA fragility in leukocytes induced by radiation exposure of Gunn and Wistar rats [39] was significantly reduced when compared to the Wistar rats. On the contrary, DNA fragility was significantly higher in Gunn rats that were not irradiated. This could be accounted either to higher DNA damage itself or might indicate differences in efficiency of DNA repair [39,40].

Groups, where UCB was administered had not only smaller spleen, liver and thymus, but they had also lower body weight and smaller hind paw volumes (Figure 1a,b). This general unwanted growth retardation was present even though rats had the same access to food and better mobility due to suppressed clinical signs of AIA. As was previously observed in mice, bilirubin administration for 14 days caused reduction of the liver size and body fat, decrease of total plasma cholesterol, insulin and leptin and raise of adiponectin [41]. In human studies, hyperbilirubinemia correlated in subjects with lower BMI and was considered as a possible factor for weight loss in obese people [39,42]. In healthy mice, UCB administration, besides significant reduction of the spleen weight, resulted in decreased viability of bone marrow cells resulting in significant decrease of leukocytes, lymphocytes and hemoglobin levels [26]. These changes seem to be specific due to UCB administration.

Despite the ongoing therapy of patient with RA some clinical studies have shown increased markers of OS in these patients [43,44,45]. The systemic autoimmune disease RA is characterized by increased cardiovascular mortality and morbidity and is an independent cardiovascular risk factor [46]. Oxidative stress has been linked to functional and structural cardiovascular alterations in animal models of chronic arthritis and in patients with RA, suggesting a complex interplay between oxidative stress, autoimmune response and inflammation in the development of atherosclerotic cardiovascular disease in RA [47]. Reactive oxygen species and reactive nitrogen species are highly reactive chemical compounds that have the potential to damage lipids, proteins and DNA favouring expression of neoantigens and initiation of autoimmunity in predisposed individuals. Accordingly, exaggerated reactive oxygen species formation and increased levels of markers of protein and lipid oxidation have been reported in several systemic autoimmune diseases, including RA [48,49]. However, there is no standard therapeutic approach to address the OS in RA patients.

To objectify the biological impact of unconjugated bilirubin administration it is important to interpret the results depending on the clinical context. In healthy animals the effect of UCB may be perceived as unwanted side effect, but in animals with AIA, the effects employ as therapeutic.

## 4. Material and Methods

### 4.1. Animals and AIA Experimental Model

31 adult male Lewis rats weighing 160–180 g were obtained from Department of Toxicology and Laboratory Animal Breeding, Centre of Experimental Medicine, SAS, Dobrá Voda, Slovak Republic (SK CH 24016). The rats had free access to the standard pellet diet and tap water as well as dark/light regime 12 h/12 h. The experimental protocol was approved by the Ethics Committee of the Institute of Experimental Pharmacology and Toxicology, Center of Experimental Medicine SAS in Bratislava, Slovakia, (3144/16-221/3, 1.1.2011) and by the Slovak State Veterinary and Food Administration of the Slovak Republic, Bratislava in accordance with the European Convention for the Protection of Vertebrate Animals Used for Experimental and Other Scientific Purposes and with Slovak legislation.

### 4.2. The Design of AIA Experiment

The animals were randomized into four groups: healthy not treated animals as Control group (CO, *n* = 7), healthy animals with UCB administration (CO-BIL, *n* = 8), untreated adjuvant-induced arthritis group (AIA, *n* = 8) and adjuvant-induced arthritis group administrated with UCB (AIA-BIL, *n* = 8). AIA was induced by single intradermal shot with suspension of 0.1 mL of 12 mg/mL heat-inactivated *Mycobacterium butyricum* powder suspended in incomplete IFA (Difco Laboratories, Detroit, MI, USA) applied to the rat tail root region according to our previous experimental protocol [50,51,52]. Therapeutic treatment regime was as follows: UCB was administered 200 mg/kg of body weight daily i.p. from day 14, to the day 28 of the study. UCB (porcine origin, AppliChem, GmbH, Darmstadt, Germany) was stored at −20 °C until reconstitution in 0.2 M sodium hydroxide to obtain water soluble sodium salt of UCB. This solution was neutralized by 1.0 M hydrochloric acid and diluted by saline for desired concentration of 50 mg/mL. Reconstituted solution was stored at room temperature in a dark place. Handling and manipulation with UCB were under low light condition to reduce its depletion. Body weight of rats was measured regularly to calculate the precise application of doses. The body weight of the animals was measured daily. The changes in body weight are shown as weight gain (g). Weight measured on the day (*n*—day 14, 21 and 28) minus weight measured on day 1. HPV was calculated as the percentage increase of the hind paw of each animal, compared to the HPV measured at the onset of the experiment by means of an electronic water plethysmometer (UGO BASILE, Comerio-Varese, Italy). The HPV was measured at the same days as change in the weight.

### 4.3. Blood Tests and Oxidative Damage Evaluation

Changes in the blood count were evaluated on 14th, 21st and 28th day of the experiment using ABX Pentra 60 analyzer (Horiba Medical, Tokyo, Japan). On 28th day, when the experiment was terminated, blood and the plasma were collected and evaluated: levels of conjugated and unconjugated bilirubin using automatic analyzer Advia 2400 (Siemens AG, Munich, Germany), oxidative DNA damage was determined by the Collin’s comet method, CRP by commercial ELISA kit (Immunology consultant laboratories, Inc., Portland, OR, USA) and ceruloplasmin by the method according to Pribyl [24,53,54]. All plasma samples were stored at −80 °C until biochemical analysis.

Single cell electrophoresis (comet assay) [52] was used to evaluate oxidative damage to DNA in lymphocytes isolated from the whole blood collected on 28th day. The levels of the marker for DNA oxidative damage, 8-oxoguanines, were calculated from formamidopyrimidine DNA glycosylase (Fpg) sites, represented by total damage (TD) values reduced about buffer score, where, in TD, *i* is a class of damage and N is the number of cells in each class:

$$\mathrm{TD}=\overset{4}{\underset{i=0}{\sum }}i.N_{i}$$

using the calibration curve y = 134.97x + 7.0612, where y refers to the number of Fpg sites and x refers to breaks of DNA [55]. The concentration of 8-oxoG per 10^6^ guanines was calculated as previously described [55]. The experiments were done in duplicate.

### 4.4. Histology and Histochemistry

At the end of the experiment, thymus, spleen, liver and hind paw knee joints were collected, weighted and processed for histological evaluation. The hind paw knee joint samples were after 24-h fixation in 4% formaldehyde decalcified in EDTA, routinely processed by embedding in paraffin, cut in 5 µm thick slices, stained with hematoxylin and eosin and evaluated by light microscopy (Leica DM2000, Wetzlar, Germany). Staining with naphtol-AS-D-chloroacetate for the detection of CHAE activity was performed to evaluate tissue infiltration by granulocytes. Conventional dyeing with PTAH was performed to evaluate the presence of fibrin in joints.

### 4.5. Morphometry and Statistics

Granulocyte infiltration and fibrin deposition in the knee joint samples were evaluated by morphometry using ImageJ 1.38 (National Institute of Health, San Diego, CA, USA) in 10 randomly selected microscopic fields at 40× magnification. Using digital color extraction, the proportion of selected positivity to the total area of tissue was assessed. The measured values were statistically evaluated by GraphPad Prism (GraphPad Prism, San Diego, CA, USA).

The data were expressed as arithmetic mean ± SEM, with 7–8 animals in each experimental group. AIA and CO-BIL groups were compared with CO (*) and AIA-BIL group was compared with AIA (+). For significance calculations, unpaired Student’s *t*-test (two sample, unequal variance) was used with the following significance designations: extremely significant (*p* < 0.001), highly significant (*p* < 0.01), significant (*p* < 0.05); not significant (*p* > 0.05).

## 5. Conclusions

Dysregulation of the innate and adaptive immune responses occur at different stages of RA development. The inflammatory process lead to functional and structural changes of joints and internal organs. According to our findings, induced systemic unconjugated hyperbilirubinemia might have beneficial effects on the course of AIA. By administration of UCB we observed improved clinical signs, decrease of inflammation and protective role in DNA oxidative damage in studied animals. To our knowledge, this is the first study that documents complex changes of blood elements variables in the time course during high dose of UCB administration in healthy animals as well in AIA for the period of 14 days.

Our research is focused to mitigate the OS in experimental arthritis, by means of substances with known and/or unknown antioxidative properties. From our perspective we think that lowering of the OS could be a part of a more complex therapeutic approach of patients with RA, together with newer agents, which are based on blocking/modifying specific steps in the inflammatory cascade. Supplementation with substances able to reduce the OS could be a part of observational clinical studies with standard anti-rheumatic treatment.

These findings might be the basis for further research on immunomodulative properties of UCB.

## Figures and Tables

**Table 1:** Changes in erythrocytes (number of erythrocytes, hemoglobin content and corpuscular volume) parameters during the course of the experiment. MCV—mean corpuscular volume. CO—control group, CO-BIL—control group administered with UCB 200 mg/kg of body weight daily i.p. from day 14, AIA—group with adjuvant induced arthritis, AIA-BIL—group with adjuvant induced arthritis administered with UCB 200 mg/kg of body weight daily i.p. from day 14. Results are expressed as mean ± SEM, n = 7–8. Significant difference between groups CO and CO-BIL, CO and AIA: *** *p* < 0.001 vs. CO, ** *p* < 0.01 vs. CO, * *p* < 0.05 vs. CO. Significant difference between groups AIA and AIA-BIL: ^++^ *p* < 0.01 vs. AIA, ^+^ *p* < 0.05 vs. AIA.

			Day					
		Group	14		21		28	
Erythrocytes (10^6^/μL)		CO	0.84	±0.04	0.96	±0.03	1.00	±0.03
		CO-BIL	0.94	±0.03	0.99	±0.13	0.96	±0.02
		AIA	0.86	±0.02	0.96	±0.03	1.05	±0.02
		AIA-BIL	0.84	±0.03	0.91	±0.02	0.92 ^++^	±0.03
Hemoglobin (g/dL)		CO	1.37	±0.05	1.63	±0.06	1.66	±0.04
		CO-BIL	1.69 **	±0.07	1.63	±0.03	1.56*	±0.02
		AIA	1.38	±0.03	1.51	±0.04	1.60	±0.04
		AIA-BIL	1.31	±0.04	1.40	±0.04	1.42 ^+^	±0.06
MCV (μL)		CO	47.71	±0.84	46.86	±0.77	47.43	±0.72
		CO-BIL	46.63	±0.32	44.63 *	±0.26	44.71 **	±0.29
		AIA	45.00 **	±0.27	43.88 **	±0.35	42.86 ***	±0.40
		AIA-BIL	43.88 ^+^	±0.40	42.86	±0.40	42.67	±0.33

## Data Availability

Data are available using this link: https://drive.google.com/file/d/1Y8DYN_CRyJ9Nka41kSFKgo0ExvTp2P88/view?usp=sharing (accessed on 12 August 2021).
